# Frequency Selective Auto-Encoder for Smart Meter Data Compression

**DOI:** 10.3390/s21041521

**Published:** 2021-02-22

**Authors:** Jihoon Lee, Seungwook Yoon, Euiseok Hwang

**Affiliations:** 1 School of Electrical Engineering and Computer Science, Gwangju Institute of Science and Technology (GIST), 123 Cheomdangwagi-ro, Buk-gu, Gwangju 61005, Korea; zoazoa61@gist.ac.kr; 2 School of Mechatronics, Gwangju Institute of Science and Technology (GIST), 123 Cheomdangwagi-ro, Buk-gu, Gwangju 61005, Korea; ysw1207@gist.ac.kr

**Keywords:** data compression, smart meter, auto-encoder, digital signal processing

## Abstract

With the development of the internet of things (IoT), the power grid has become intelligent using massive IoT sensors, such as smart meters. Generally, installed smart meters can collect large amounts of data to improve grid visibility and situational awareness. However, the limited storage and communication capacities can restrain their infrastructure in the IoT environment. To alleviate these problems, efficient and various compression techniques are required. Deep learning-based compression techniques such as auto-encoders (AEs) have recently been deployed for this purpose. However, the compression performance of the existing models can be limited when the spectral properties of high-frequency sampled power data are widely varying over time. This paper proposes an AE compression model, based on a frequency selection method, which improves the reconstruction quality while maintaining the compression ratio (CR). For efficient data compression, the proposed method selectively applies customized compression models, depending on the spectral properties of the corresponding time windows. The framework of the proposed method involves two primary steps: (i) division of the power data into a series of time windows with specified spectral properties (high-frequency, medium-frequency, and low-frequency dominance) and (ii) separate training and selective application of the AE models, which prepares them for the power data compression that best suits the characteristics of each frequency. In simulations on the Dutch residential energy dataset, the frequency-selective AE model shows significantly higher reconstruction performance than the existing model with the same CR. In addition, the proposed model reduces the computational complexity involved in the analysis of the learning process.

## 1. Introduction

Increasing interest in ecofriendly and sustainable power usage has changed the paradigm of energy management [1] from a supply-based to a demand-oriented policy that encourages efficient energy usage by consumers, in view of their continued efforts to create new industries. Convergence of industries is accelerating with the advent of internet of things (IoT) technology, while big data collected through various devices is essential for creating new opportunities and values. Energy services provided by the combination of IoT technology and big data are evolving into intelligent services and their related smart objects [2]. Thus, the importance of incorporating information and communication technology (ICT) with the existing technology in the smart grid is increasing. Specifically, a smart meter is a necessary platform in the advanced and active management systems of energy data information [3]. As smart metering systems are being installed on a large scale worldwide, they must process large data through numerous nodes connected in a network [2]. In addition, the quality of services delivered to consumers and the performance of various applications, such as dynamic pricing [4], demand side [5], management [6], and load forecasting [7,8] could be improved by improving smart metering systems [9,10,11,12]. The energy data generated by smart meters are characterized by a high sampling rate and periodic collection. In addition, the high sampling data generated by smart meters is used in various research fields, such as non-intrusive load monitoring (NILM), user segmentation, and the analysis and prediction of power consumption patterns. Improved performance can be confirmed by downsampling based on the micropatterns in the high-sampling data [13]. The data sampling rate of the sensor installed in a smart meter is provided in the specifications and is not fixed; accordingly, the size of the generated data reaches several hundred terabytes per year [14]. However, an excessive amount of data causes additional burdens such as traffic problems related to the transmission process of the channel from the advanced metering infrastructure (AMI) to the cloud server. Therefore, compression and data pruning technique for efficient transmission is becoming more important to save the bandwidth and the storage cost [2]. In addition, a generalized structure of the compressed and pruned data is needed to communicate with other agents in the grid in an efficient way. In this aspect, additional compression of the data is possible according to the correlation structure of the generated data from the user’s power consumption or other agents in the community [15].

The compression technology is divided into lossless and lossy compression technologies. There is a trade-off between the compression ratio (CR) and the information loss of the data, depending on the purpose of usage of the two compression techniques [16]. Lossless compression is used for high-resolution or nonpersistent data with a nonvisible pattern. Representative lossless compression methods include entropy coding, dictionary coding, and deflate coding [17]. In [18], a lossless compression method that compresses the waveform approximated with few using the Gaussian approximation is proposed. Lossy compression is applied to error-tolerant scenarios, such as the case where a high CR is needed or a wireless communication environment [2]. In addition, lossy compression is mainly used as a method for efficiently compressing sensor data such as smart meter data to primarily reduce the cost related to the limited aspects of bandwidth, energy, and storage in the transmission area of the IoT environment [18]. Lossy compression methods include discrete wavelet transformation (DWT) [19], singular value decomposition (SVD) [20], principal component analysis (PCA) [21], and compressive sensing (CS) [22]. In addition, a compression method, which captures the interdependence of multi-variables generated by a smart meter, using PCA and CS has been recently proposed [2]. A compression method based on deep learning techniques such as an auto-encoder (AE) has been additionally proposed [23].

For AE, compression, sparsity or another data structure is not required; instead, the appropriate structure is learned from the data within the range of the allowed compressions using nonlinear techniques. In addition, it can be argued that it shows better performance than other linear operation-based compression methods such as PCA since AE applies a neural network (NN) to select an appropriate nonlinear activation function that performs a nonlinear operation on the data [24]. Moreover, nonlinear transformation, multiplication, and summation are included in the process to reconstruct the data efficiently. Thus, it secures the possibility of generalization of the trained model when applied to other datasets [25]. However, experiments confirmed that the AE compression method had an adverse effect on the reconstruction due to severe fluctuation in the data collected with high sampling. In addition, most differential coding-based compression techniques tend to be less efficient because they are sensitive to small differences in consecutive values of smart meter data. The reason is that compressing household power data of high resolution in smart meters is a real challenge due to the rapidly changing load patterns [23,26]. In [23], a method of selectively operating a customized compression model of each signal was proposed to improve efficiency. The stacked, sparse, variational, and convolutional AE models [27], which are commonly used models were applied to the Dutch residential energy dataset (DRED) [28], one of the public datasets of energy to investigate data-dependent characteristics. Results of the analysis show that the reconstruction performance is excellent for simple waveforms such as pulses. On the other hand, waveforms with high-frequencies such as noise signals showed high errors due to fluctuation or nonperiodicity, resulting in reduced reconstruction performance.

This paper proposes a compression structure that reflects the data-dependent features of smart meter data and aims to achieve efficient compression through a lightweight model such as the AE model, to improve the low reconstruction quality checked to severe fluctuation. After analyzing the frequency characteristics of smart meter data through a signal processing technique, data is separated and a deep learning-based compression method is applied. First, to improve the reconstruction performance, the proposed method separates the frequency domain based on a specific threshold in the power spectral density (PSD) [29] transformation. Using this, it is verified that the training process of the deep learning model has an advantage by grouping data with similar frequency characteristics. Moreover, the performance is improved when data is reconstructed at the same CR as the existing method. Second, it is confirmed that the computational complexity was reduced by analyzing the learning process of the NN model applied in the proposed method. As a result of checking the learning curve of the model, it is evaluated to confirm faster convergence than the existing method. A variety of AE models are applied to the proposed method to verify the effectiveness of the proposed method.

Section 2 introduces the features of smart meter data, signal processing techniques applied to the proposed method, and types of various compression techniques. Section 3 presents the data preprocessing and specific network architectures used in the proposed method. In Section 4, the results of the feasibility test applied to the proposed method, and the compression performance of the benchmark model is provided. Through this, the applicability and effectiveness of the proposed method can be confirmed. Finally, conclusions are summarized in Section 5.

## 2. Backgrounds

### 2.1. Spatio-Temporal Compression for Smart Meter Data

Smart meters collect and transmit various data such as active power, reactive power, voltage, and current with a time stamp. Measured data are compressed and stored in the internal buffer, because their sampling rate can be as high as 12–250 kHz [30]. Temporal or spatial compressions can be applied on power data, and spatio-temporal compression is also feasible [26]. Temporal compression is applied for the data of each node on the smart meter such as CS, which compresses smart meter data, considering data sparsity in the time domain. It is possible to reduce the burden on the communication channel transmitted to the data control center through the compression process performed in each node. Alternatively, spatial compression [31] can be applied to multiple nodes by taking advantage of the spatial correlations of similar patterns in distributed nodes such as PCA compressing data into low-dimensional space (principal components) that do not have a linear relationship. Using spatial correlation methods, the data collected from individual meters are transmitted to the local data concentration unit (DCU) and compressed before being transmitted to the central control center. Spatio-temporal compression is a method jointly considering temporal and spatial compression. Since the power data shows correlations along both spatial and temporal direction, spatio-temporal compression may better perform than individual temporal or spatial compression models. It is suitable to be employed as a compression model in the smart grid environment. However, it should be noted that spatial compression can be processed only when the size of the dimension of the data compressed by the previous step, temporal compression, are equal.

### 2.2. Short-Time Fourier Transform and Power Spectral Density

The short-time Fourier transform (STFT) [29] is a sequence of Fourier transforms of signals within individual time windows. The Fourier transform gives the averaged frequency information over the entire time interval while, STFT provides time-localized frequency information in an environment where the frequency component of a signal changes over time. STFT can be expressed as follows:

(1) $$X\left[n,\lambda \right)=\sum_{m\;=\;-\infty }^{\infty }x\left[n+m\right]w\left[m\right]e^{-j\lambda m}$$

(2) $${X\left[n,k\right]=X\left[n,\lambda \right)|}_{\lambda \;=\;\frac{2\pi k}{N}}$$

where X[n,λ) denotes the STFT of the *m*-shifted signal, x[n+m], viewd through the window sequence, w[n]. Here, *n* and λ are the discrete time and frequency variables, respectively, which range from 0 to 2π. STFT has a trade-off between windowed time and frequency resolution. Specifically, a narrow window produces a better resolution in the time domain but a lower resolution in the frequency domain. The opposite is also true. PSD expresses the energy of the time signal in the frequency domain, which is applicable to nonperiodic signals or when integration of the squared signal is difficult to calculate. The output of the stationary state is sufficient for calculating the PSD values of the time signal. The PSD is determined as follows:

(3) $$S\left[n,k\right]=\log{\left|X[n,k]\right|}^{2}$$

where S[n,k] denotes the PSD value of the *k*-th window, obtained by taking the value of the squared STFT on the logarithmic scale.

### 2.3. Auto-Encoder for Data Compression

Generally, the NN has an input **x** and an output **y**. It can be used to develop a model that regresses **x** with respect to **y**, as follows:

(4) $$y=f_{NN}\left(x\right)$$

In other words, the output **y** is generated using a multi-layer structure $f_{NN}\left(\cdot \right)$ for the input **x**.

Generally, transformation is a complex nonlinear function obtained by the construction of an activation function, f(x), for successive layers in a network. Among the various structures of NN, the AE model [25,32] has a pair of activation functions, an encoder, and a decoder. The encoded latent vector provides the desired features, and the decoded vector is designed to be the same as the input of the encoder as follows:

(5) $$z=f_{enc}\left(W_{1}x+b_{1}\right)$$

(6) $$\hat{x}=f_{dec}\left(W_{2}z+b_{2}\right)$$

where **z** denotes the latent vector extracted by the encoder, $f_{enc}\left(\cdot \right)$, and the reconstruction, $\hat{x}$, is generated by the decoder, $f_{dec}\left(\cdot \right)$. Note that for compression, **z** must have a shorter length than **x**. The components of the encoder and decoder comprise an activation function, a weight term **W**, and a bias term **b**. As a nonlinear representation of the identity function (or matrix) connecting the input and output, the AE model learns a nonlinear function as the output from the hidden layer related to the input. The AE model is a lossy compression model that aims to minimize the mean absolute error (MAE) or the mean squared error (MSE) between **x** and $\hat{x}$, as follows:

(7) $$\min({\mathrm{loss}}_{MAE}=\sum_{i\;=\;1}^{n}\frac{|x_{i}-{\hat{x}}_{i}|}{n})$$

(8) $$\min({\mathrm{loss}}_{MSE}=\sum_{i\;=\;1}^{n}\frac{{\left(x_{i}-{\hat{x}}_{i}\right)}^{2}}{n})$$

where $x_{i}$ denotes the *i*-th sample in input x. With these characteristics, the AE model is able to serve the role of dimension reduction and feature extraction algorithms for unsupervised learning.

**Figure 1:**
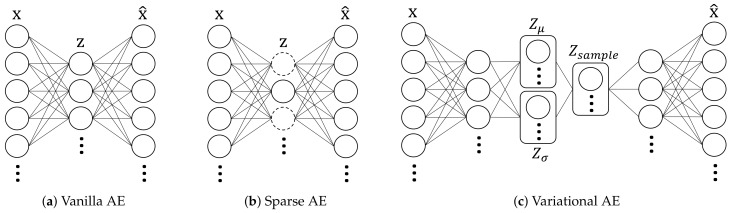
Illustrations of auto-encoder (AE) models.

Related to representative types of AE compression models [33,34], there are vanilla [35], sparse [36], and variational [37] AE model. The vanilla AE structure is a basic AE model, which is composed of an encoder and a decoder network as shown in Figure 1. An encoder is a part of the NN structure that performs a compression process, while the decoder performs the decompression process. It is important that there is a difference in the role of converting real numbers to bit strings like existing encoder and decoder blocks. The AE encoder is a NN structure composed of a multi-layer neural network, modeling the connection between the input and the latent vectors, which is the compressed data. In the case of the AE decoder, a multi-layer NN structure is constructed by modeling the connection between the latent vector and the output. In addition, the AE model is usually used in a structure with symmetric encoder and decoder, based on a latent vector.

The sparse AE model can take advantage of the internal structure to the input by applying a sparse constraint and KL-divergence [38]. In other words, additional normalization, which is a sparse constraint for hidden units, has been developed to utilize the internal structure of the data. Neurons with output close to one are activated and neurons with output close to zero are deactivated. The sparse AE model aims to limit neurons to be inactive most of the time.

The variational AE model is a recently developed AE model that is concretely classified as a generative model. Unlike the output of a traditional AE model, the outputs of the encoder and decoder represent the samples taken from a parameterized probability density function (PDF). As shown in the structural features of the variational AE model, it consists of an encoder that is a generative model and a decoder that is a recognition model. This allows the parameters of the model to be sampled from a specific statistical distribution. In addition, the variational AE model imposes constraints on hidden neurons. In terms of coding theory, this hidden neuron can be interpreted as a latent vector or code. The NN structure of variational AE model itself can balance the reconstruction accuracy and the goodness of fit of the Gaussian distribution. In summary, the variational AE model is characterized by a parameterized distribution of the prior probabilities in the compressed representation, and the parameters are learned by the AE structure. This model has been recently utilized for compression of image data [39].

## 3. Proposed Methods

### 3.1. Network Architecture and Specifications

**Figure 2:**
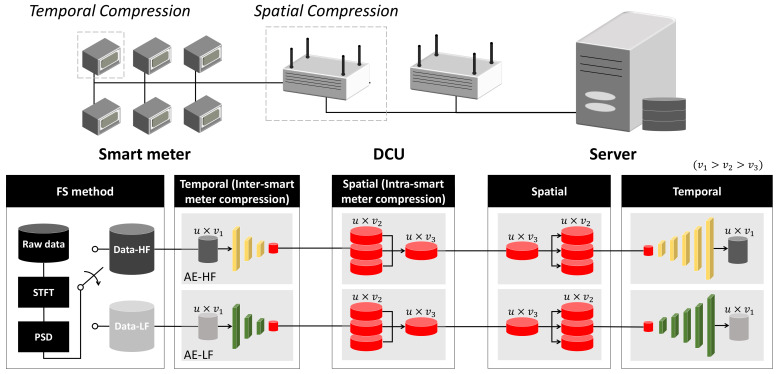
Overall diagram of the proposed method.

Figure 2 shows the framework of the proposed method. The data size d is determined as u×v, whereas the original, temporal, and spatial compressed data are represented by $d_{1}$, $d_{2}$, and $d_{3}$, respectively. As the characteristics of smart meter data are widely varying over time, multiple models customized for specific properties may need to be switched for efficient compression. In particular, the collected waveforms are divided into multiple fixed time windows where different compression models can be trained and applied, depending on the spectral properties of the windows.

For example, based on PSD analysis, the windowed waveforms can be divided into two clusters, high- or low-frequency dominant signals. In the server, AE compression models for high-frequency (AE-HF) and low-frequency (AE-LF) data are generated. The encoder of the temporal compression model is then transmitted to the smart meter, and the decoder used for data reconstruction is stored on the server. In this way, the data collected by individual smart meters are transmitted to and self-compressed in the DCU. The compressed data are transmitted and stored on the server through the DCU whereby spatial compression is additionally conducted. When data reconstruction is needed, it is restored by the corresponding decoder, which can be detected from the latent variables. The server calculates correlation values between transmitted and reconstructed data using decoders, thereby selecting a decoder for reconstruction. Throughout the paper, the frequency response-based pair of the two models is investigated, though multiple models can be accounted in the same manner with a trade-off between compression efficiency and additional complexity in training and detecting.

### 3.2. Frequency Selection (FS) Method

**Figure 3:**
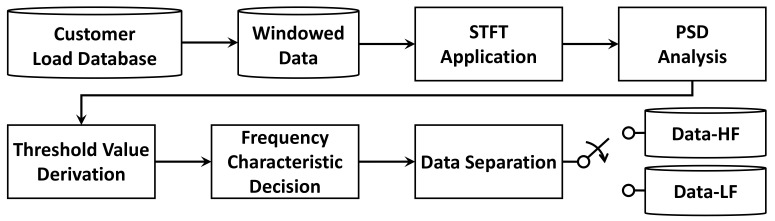
Specific diagram for the frequency selective method.

To reduce error when reconstructing compressed data of high-frequency data, the FS method is applied to a signal processing technique to separate the data into high- and low-frequency sections as shown in Figure 3. The training data separated through the FS method contributes to improve the reconstruction quality because it is applied advantageously during the learning process of each AE model to generate a compression model. The proposed method uniformly divides the entire section of the training data by a specific window size. The domain of each window is then converted from time to frequency using the STFT technique. To determine the threshold according to the magnitude of the power signal of each window in the frequency domain, PSD technique is applied, which indicates the size of the signal and determines the area with large fluctuation. To classify a signal exhibiting a large fluctuation, a threshold value of PSD is set. Concretely, the data is separated by setting the region above the threshold value as a high-frequency window and that below the threshold value as a low-frequency section. Thereafter, an AE compression model suitable for each characteristic of the data separated from the high- and low-frequency windows is trained.

### 3.3. Auto-Encoder Compression

**Figure 4:**
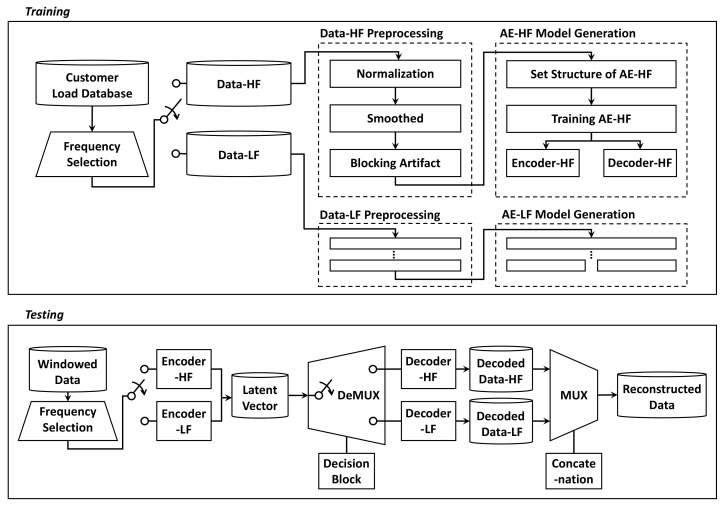
Application to the auto-encoder compression.

The dimension $v_{2}$ of the latent vector for compression is a parameter that can be set by the user when configuring the AE compression model. In this case, it can be made to learn the desired data representation, according to the size of $v_{2}$. When $v_{2}$ is larger than the dimension $v_{1}$ of the original data, it is expressed as an overcomplete structure. The opposite case is defined as an undercomplete structure. For example, an overcomplete AE model can be treated as a signal having sparsity to learn a sparse representation of input data for a higher dimensional space. On the other hand, the undercomplete AE model is applied for representation of data compression, which is dealt with in this paper. The compressed data, which is smaller than the original data, can be stored as a low-dimensional latent vector of the NN structure. In general, as the size of the compression vector $v_{2}$ increases, the compression performance decreases, but the reconstruction performance improves. This property indicates an inverse relationship between the two indicators [25]. AE compression models are used to learn data separated into high- and low-frequency sections. It generates an AE compression model that reflects the frequency characteristics of each window region, respectively. Through the proposed method as Figure 4, the reconstruction error rate of compressed data in a window region with severe fluctuation is reduced using the learned model. The parameters of the vanilla AE model implement the experiment of the proposed method referred to [25], and tuning is performed for optimization.

## 4. Experiments and Results

### 4.1. Experimental Setup

In this paper, it is experimentally confirmed the structure of the AE model optimized for the proposed method to calculate the PSD values for time signal. Moreover, we confirmed the effects of data preprocessing, such as moving averaged (smoothed) and block artifacts. In addition, the validity of the proposed method is verified by applying different types of AE compression models such as sparse and variational AE model under the same conditions with vanilla AE model. Lastly, decoders can be automatically selected through the compressed latent vector for a practical point of view, which was systematically designed by utilizing the correlation of the latent vector used in the learning process. Based on the results, threshold is set through the process of classifying by applying the machine learning model. Using the threshold, the compressed latent vector and decoder having the same frequency characteristics are selected.

This paper assumes that one DCU is connected to several smart meters for data transmission. The bandwidth requirement for this situation is evaluated [40]. Specifically, when the individual smart meters are directly connected to the DCU, the sampling and transfer intervals are assumed as 1 second and 4 minutes, respectively. When a single smart meter transmits the power consumed by the entire household, the average bandwidth requirement is 1401.8 bps/AMI. The CR in the experiment was approximately 20%, implying that the bandwidth can be reduced by approximately 80%; equivalently, the amount of transmitted AMI data can be increased by a factor of 5.

To evaluate the proposed compression method, the public dataset is employed. DRED dataset, which is one of the NILM datasets, is used for the case study. The DRED dataset has aggregated power data of residential level and includes a 6-month period with 1 Hz resolution. The result is obtained by simulating 375–475 k sample points using the power data of the DRED dataset. The AE model for the experiment had a fixed NN structure with a single layer and 256 neurons. The optimizer and the activation function used for training the AE model were applied to the ADAGRAD algorithm along with a parametric rectified linear unit (PReLU) for activation. During the learning process of the AE, 80% and 20% of the experimental data were randomly split into the training and test sets, respectively, using the 5-fold cross validation [25]. To the encoder of the sparse AE, we added an L1 regularizer, which imposes a cost constraint proportional to the absolute value of the weight. In the experiments, the cost was set to 0.0001. In the variational AE, unlike the previous two models, the activation function for the encoder was a rectified linear unit (ReLU). In this model, a cost proportional to the square of the weight was imposed, using the weight decay method. For the learning process, the experimental value was set to 0.003. The vanilla, sparse, and variational AE models were run in the same experimental environment. The reconstruction error rate of the following equivalent CR was equally evaluated for each model:

(9) $$\mathrm{CR}\;=\;(\frac{\mathrm{Compressed}\mathrm{size}}{\mathrm{Original}\mathrm{size}})\times 100$$

As shown in Table 1, the vanilla AE model delivered the best performance among the three models. Thus, the vanilla AE model was optimized in subsequent experiments and applied to a feasibility test of the FS method.

In the proposed method, the negative effects of unpredictable event signals were reduced by moving-average smoothing. This step reduces the noise signals in the training data, from which the features are extracted. By mitigating the influence of the event signal within the range of a specific window, it aims to minimize the reconstruction error rate. The reconstruction quality is further improved by converting the data to a matrix form and applying techniques for blocking artifact. The technique for blocking artifact is related to data overlap, and its process is described [2,15,25,41]. The window showed a clear pattern over increments of approximately 4 minutes. Thus, 256 samples per window were taken for a convenient fast Fourier transformation (FFT) conversion. The overlap length was 130 samples, approximately half the window size. To evaluate the usefulness of these data preprocessing steps, smoothing and blocking artifacts were applied to the vanilla AE model, without changing the model parameters. The results are given in Table 2. The reconstruction quality of the compression model was improved after combining the two pre-processing methods.

To find the optimal structure of the vanilla AE model, the reconstruction error was determined for different numbers of hidden layers (see Table 3). Conclusively, the best performance was achieved by inserting one layer in the encoder and three layers in the decoder. Therefore, this asymmetric structure was used in the following feasibility test.

### 4.2. Frequency Selective Processing

**Figure 5:**
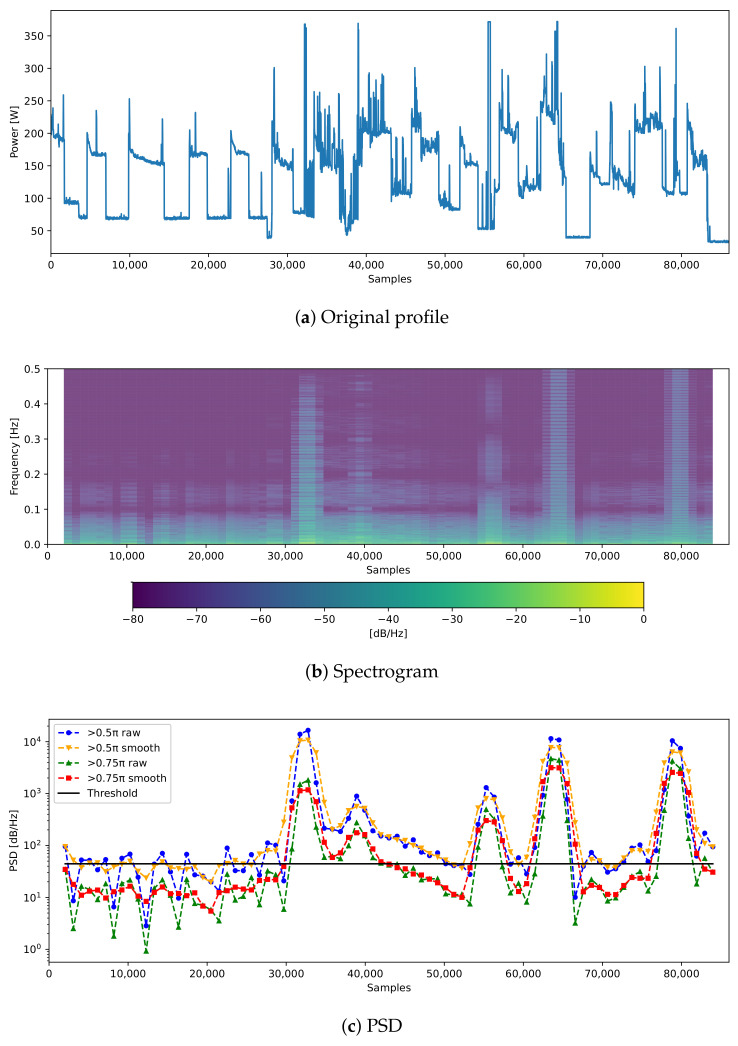
The application process of the frequency selective (FS) method.

First of all, STFT is used to change from time to frequency domain to analyze the frequency characteristics of test data. Specifically, the size of the individual window was set to 256 samples to which the Hamming window was applied. The overlap size applies to 60% of the window samples, which can show the clear frequency characteristics of the test data. Using this, high-frequency components with fluctuation can be identified when viewing this as a log-scale spectrogram. After 50% or 25% of the power of the high-frequency part is considered and added, the moving average is taken. The results can be confirmed as shown in Figure 5. A specific threshold is taken from the PSD domain checked in this way. If the PSD value is larger than the threshold, a high-frequency favorable method can be selectively applied. In this experiment, the median value of the PSD values was taken as a threshold value and classified into high- and low-frequency data.

### 4.3. Feasibility Test

The feasibility test confirms the ideal experimental results of the proposed method that is completely separated data by the FS method. For evaluating its validity, three metrics, including the information entropy, reconstruction error, and model learning process, are used.

**Figure 6:**
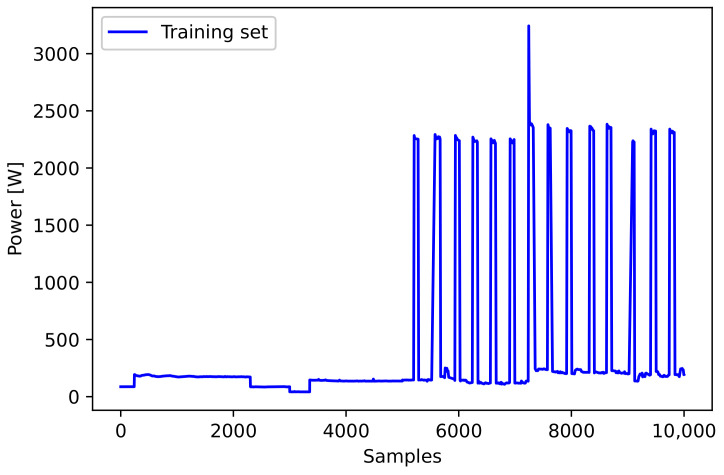
The profile of sample data for the feasibility test.

For comparing the performance of the proposed method, we used the training datasets to which the frequency selective AE (FS-AE) method was applied and not applied in the same area. Specifically, the existing and proposed methods were trained on 10k samples extracted from the DRED dataset, as shown in Figure 6. The performance results are shown in Table 4. The samples outside 3σ of the original data (12 samples) were considered as spike points and removed. Accordingly, the existing AE model included 110 reconstruction data and its MAE was 1576.13. Meanwhile, the FS-AE model included 91 samples and its MAE was 1483.02. Therefore, the loss of data due to the spike points was reduced in the proposed method. The experimental results confirmed that the proposed FS-AE method reduces the reconstruction error.

In addition, the information entropy of the original, compressed, and reconstructed data is evaluated using the index of information theory when applied to the FS-AE method. The same values of information entropy can be confirmed in Table 5, i.e., it can be confirmed that the reconstruction error can be improved without loss of additional information by applying the FS-AE method.

**Figure 7:**
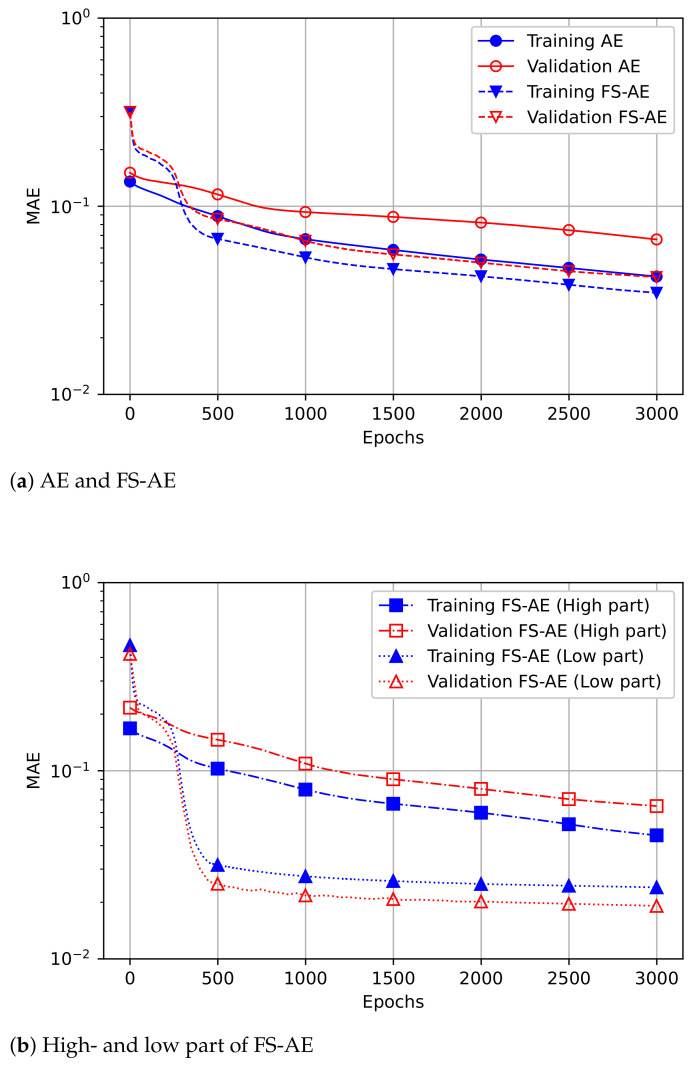
Comparison of the speed of convergence for the training and validation loss (mean absolute error (MAE)) by (**a**) the existing and the proposed method, (**b**) the high- and low-frequency part of the proposed method, each applied for half of the test data.

Regarding the learning analysis of the model, it is possible to ascertain the MAE loss according to the epochs, as shown in Figure 7. The learning curve in the low-frequency part converges at less than 500 epochs. The learning speed of the training and validation sets of the proposed method is observed to converge faster than the existing method. Through this, it can be seen that the computational complexity of the model decreases in proportion to the number of epochs in the training process of the AE model.

To evaluate the performance of the proposed method according to the number of thresholds, the threshold value is equally divided by 1/2 to 1/9 of the PSD value. Experimental results in Table 6 reveal that the performance of the FS-AE method is improved compared to the existing AE model and that the reconstruction error is also improved as the number of sections divided increases. However, the reconstruction error is not improved in direct proportion to the number of divided sections, unlike the increase of the computation in the AE compression model. Considering the efficiency of using actual models, operating a large number of models can lead to ineffective results. Therefore, it can be determined that it is most efficient to operate two AE models by setting the median of PSD values as thresholds similar to the experiments.

**Figure 8:**
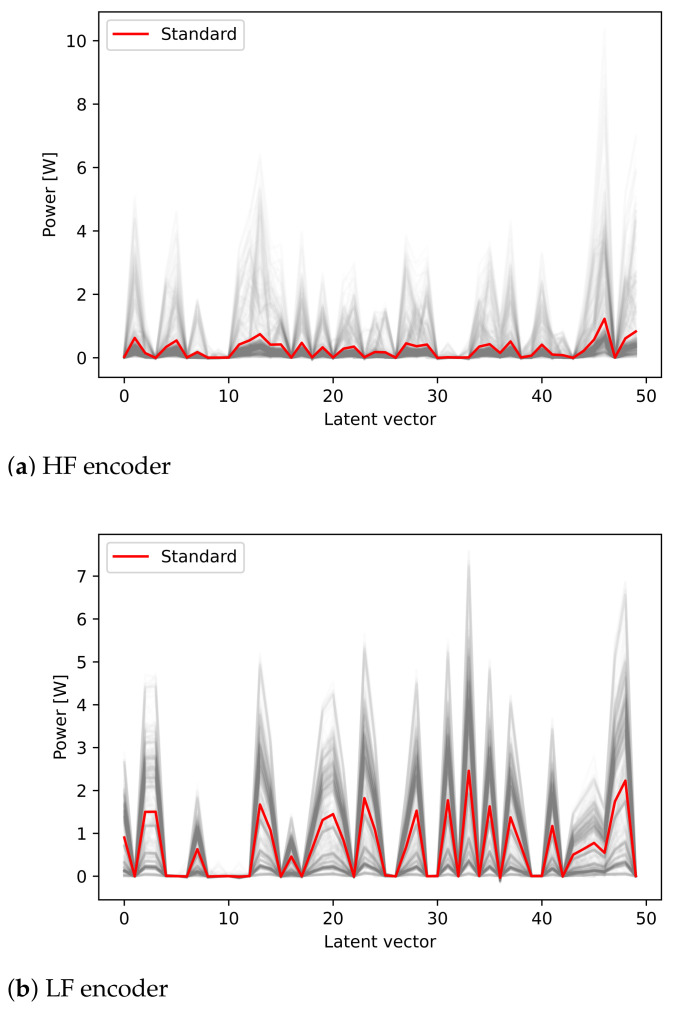
Patterns of averaged power versus latent vector after compression using the encoder.

**Figure 9:**
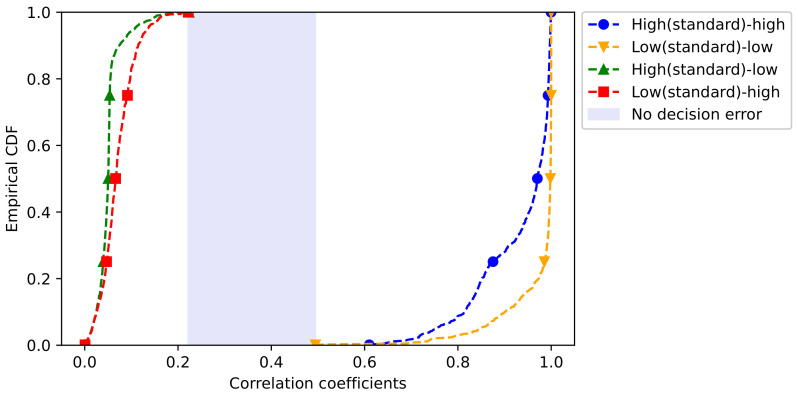
Cumulative distribution function (CDF) plot for setting the decision boundary, based on the correlation coefficients in the decoder selection.

The decoder selection test was conducted using a correlation analysis to validate if latent vectors generated by an encoder can select the same decoder. For example, it was checked whether a latent vector compressed with a HF encoder can select a HF decoder. As shown in Figure 8, it can be seen that the pattern of averaged value by each latent vector compressed using an HF or LF encoder is different. Using this as labeling information of the decoder model, we ascertained the correlation with the compressed latent vector. Specifically, four cases of HF encoder–HF decoder, HF encoder–LF decoder, LF encoder–HF decoder, and LF encoder–LF decoder, were identified, respectively. The cumulative distribution function (CDF) plot for checking the correlation confirms the high correlation at the encoder and decoder with the same frequency characteristics as shown in Figure 9. In the reverse case, it can be confirmed that there is a low correlation similar to the HF encoder – LF decoder. Based on the results of the correlation analysis, the boundary is set by the classification applied to machine learning models such as the support vector machine (SVM).

### 4.4. Scalability Test

In the experiment to confirm the scalability of the proposed model, in addition to vanilla, sparse, and variational AE models, stacked convolutional sparse auto-encoder (SCSAE) [32] model is used, which is a recently proposed technology in evaluating the applicability of the proposed method. First, the experimental results were confirmed by applying the proposed method to the AE models evaluated in Table 1. In the same experimental environment, the proposed method clearly outperformed the existing method (see Table 7). Specifically, the vanilla AE model showed the best performance, but the results were also improved by the FS-Sparse AE model. Among the three AE models, the sparse AE model gave the best reconstruction performance because it learns the sparse representation in similar training data divided by the frequency. The variational AE model, in which the modeling method is based on statistical parameters, could not adequately handle the noise signal in the high-frequency domain, hence its performance was low.

**Figure 10:**
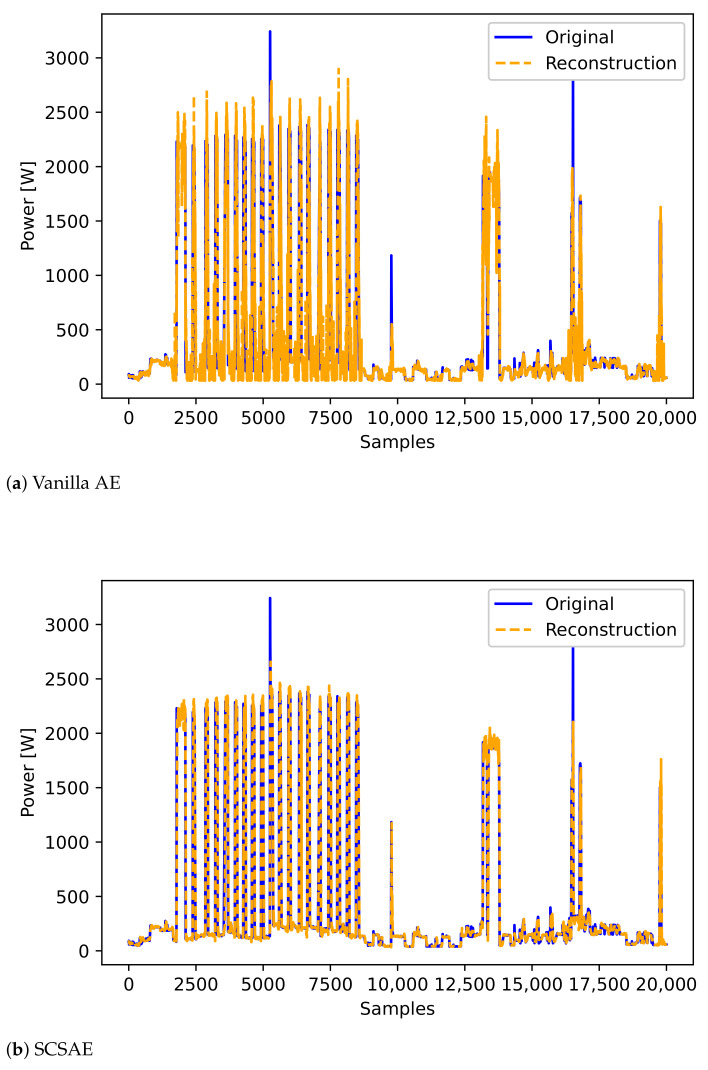
Reconstructed profile of each AE model.

In subsequent experiments, the performance of the SCSAE model was compared with that of the vanilla AE model. The parameters of the SCSAE model were set by Bayesian tuning. The SCSAE model achieved a higher reconstruction quality than the vanilla AE model (see Figure 10). The MAE loss was 84.36 for the AE model and 62.29 for the SCSAE model.

**Figure 11:**
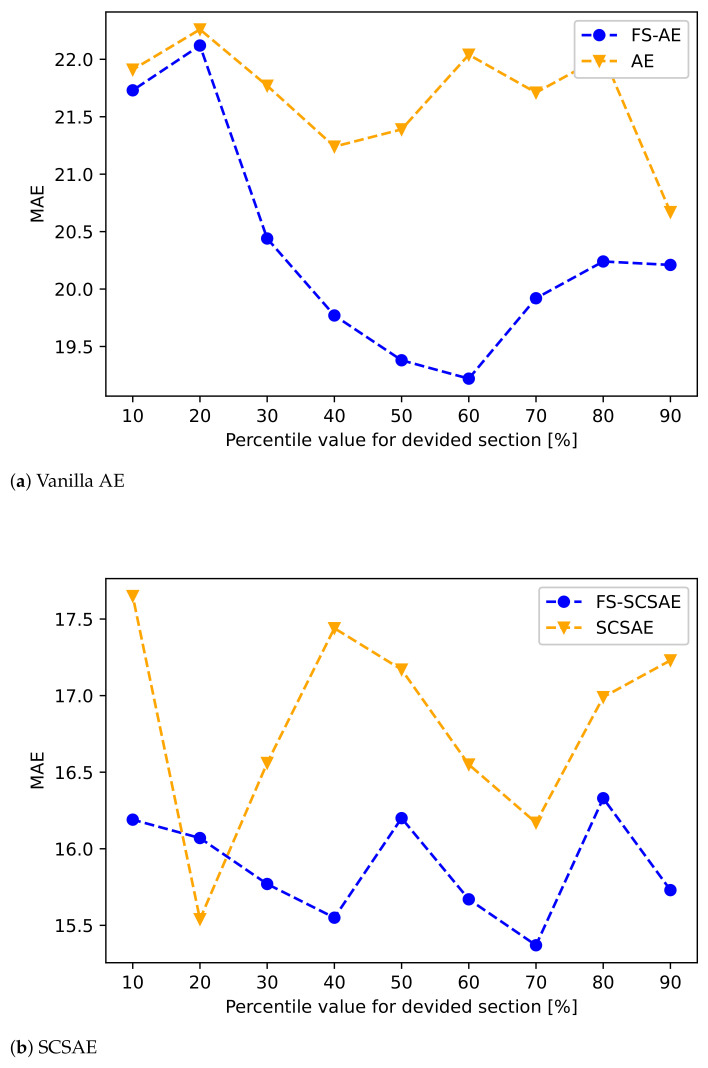
Comparison of reconstruction errors (MAEs) of the existing and proposed methods at different thresholds.

As shown in Figure 11, a comparison is made between the FS-SCSAE model applying the proposed method and the existing SCSAE model in terms of the reconstruction quality. The standard of this evaluation was the error rate of reconstructing the compressed data for different thresholds of the percentile range of PSDs. In this experiment, the thresholds were optimized. Comparing the MAE results at the optimal values, it was observed that the proposed method outperformed the existing method.

To compare the effectiveness of the proposed method, the compression algorithm, such as the kernel-PCA [42] and the truncated SVD (T-SVD) [20] was simulated in the same experimental condition. The training set is 80% of the overall data set and the test set is 20%. In the case of the kernel-PCA, the parameter of the kernel was applied for the linear. The results of evaluating reconstruction performance at the same compression ratio are summarized as shown in Table 8. As a result, the proposed method was confirmed with the best performance due to the reduction of reconstruction error in the test set.

**Figure 12:**
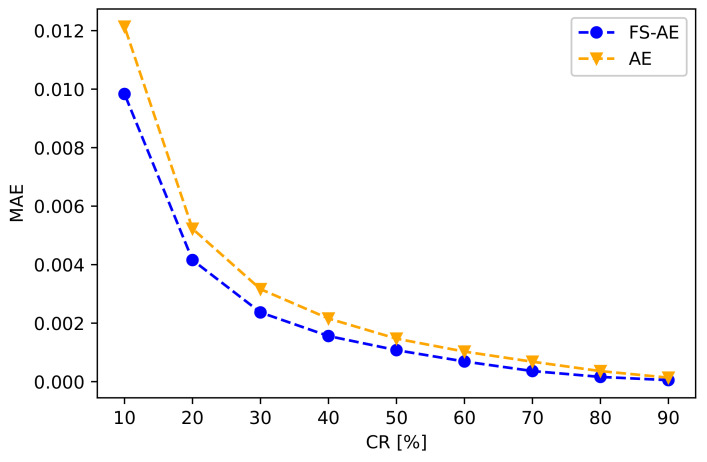
Reconstruction errors (MAEs) of the existing and proposed methods in decoding the spatial compression.

The latent vector compressed in the AE encoder model was additionally compressed to check the effect of spatio-temporal compression. The reconstruction errors in the latent vectors extracted by the proposed and existing methods were evaluated after decoding the PCA results, where the PCA was applied as a representative dimensional reduction method. The reconstruction error in the proposed method was based on the specific gravities of the high and low frequencies present in the test data. The additional spatial compression improved the performance of the proposed method, as shown in Figure 12.

## 5. Conclusions

In this paper, a novel compression method was studied to improve the reconstruction quality of the AE compression model in the smart meter environment. The FS-AE method is designed to analyze the high- and low-frequency characteristics during the training process of the AE compression model. It separates the data by reflecting the frequency characteristics to reduce the reconstruction error in the fluctuation part in the smart meter data collected at a high sampling rate. Using STFT, a threshold is set by calculating a specific PSD value that can divide the data to high- and low-frequency sections. It is intended to be favorable in constructing the AE model during training process. For the AE model selection, the usefulness of data preprocessing such as smoothing and blocking artifacts and the number of layers of NN are explored, and the asymmetry structure were tuned and confirmed. Also, the number of efficient operation intervals was confirmed considering the computational complexity proportional to the number of thresholds and models. The effect of the proposed method can be verified by comparing the reconstruction error of existing AE and proposed FS-AE models. The results can be analyzed theoretically through correlation with entropy of the latent vectors. In addition, it is possible to confirm the reduction in computational complexity in the FS-AE method. For this, we analyzed the convergence results from the compression model learning curve. The decoder selection test confirmed that the encoder and decoder models classified by the frequency characteristics can be systematically selected. In the experiment for confirming the scalability of the FS-AE method, the proposed method is simulated using the sparse and variational AE models, which are mainly used in AE compression. In addition, the SCSAE model is validated to confirm the scalability even when applying the latest technology. Lastly, through the test that performed the spatio-temporal compression, it was found that the proposed method improved the performance compared with the existing method.

## Figures and Tables

**Table 1:** Reconstruction errors for each AE model.

Model	MAE	MSE
Vanilla [35]	4.72	153.72
Sparse [36]	4.88	170.11
Variational [37]	9.05	601.31

**Table 2:** Evaluation of data preprocessing steps, based on the vanilla AE model (one hidden layer).

	Non-Overlapped	Non-Overlapped	Overlapped	Overlapped
	& Non-Smoothed	& Smoothed	& Non-Smoothed	& Smoothed
**MAE**	5.94	5.19	5.21	4.29
**MSE**	211.91	169.02	183.41	134.62

**Table 3:** Comparisons of error reconstruction rates in models with different structures.

Model Structures	Encoder Layer	Decoder Layer	Epochs	MAE
**256**→150→100→**50**→100→150→**256**	3	3		4.41
**256**→**50**→**256**	1	1	3000	3.52
**256**→**50**→100→150→**256**	1	3		3.38

Bold values: Input, latent vector, and output.

**Table 4:** The evaluation of the proposed method applied to the vanilla AE model.

	AE		FS-AE	
	Overall (10k)	Overall (10k)	High Part (10k)	Low Part (10k)
**MAE**	148.44	124.92	169.39	3.88

**Table 5:** Analysis of the information entropy to compressed latent vectors between the AE and FS-AE models.

	Original [bit]	Latent [bit]	Reconstructed [bit]
**AE**	9.9411	11.8917	13.2871
**FS-AE**	9.9411	11.8917	13.2861

**Table 6:** Reconstruction performance based on the number of separated sections.

	Threshold	Section	AE	FS-AE
MAE	one-half	[ 1/2, 1 ]	21.55	19.74
	one-third	[ 1/3, 2/3, 1 ]		19.84
	one-quarter	[ 1/4, 2/4, ..., 1 ]		19.21
	one-ninth	[ 1/9, 2/9, ..., 1 ]		18.83

**Table 7:** MAE losses in the reconstructed data for the existing AE and proposed FS-AE methods.

Model	AE	FS-AE
Vanilla [35]	4.72	4.60
Sparse [36]	4.88	5.75
Variational [37]	9.05	7.68

**Table 8:** Evaluation of reconstruction errors (MAEs) of the comparative methods.

Method	Training Set	Test Set	Overall Dataset
Kernel-PCA	9.93	49.19	17.84
T-SVD	9.94	49.11	17.83
SCSAE	18.31	9.80	16.59
FS-SCSAE	17.68	8.94	15.92

## Data Availability

Not applicable.

## Abbreviations

The following abbreviations are used in this manuscript:

AE	Auto-encoder
AMI	Advanced metering infrastructure
CDF	Cumulative distribution function
DCU	Data concentration unit
DRED	Dutch residential energy dataset
DWT	Discrete wavelet transformation
FFT	Fast Fourier transformation
FS	Frequency selective
HF	High-frequency
ICT	Information and communication technology
IoT	Internet of things
LF	Low-frequency
MAE	Mean absolute error
MSE	Mean squared error
NILM	Non-intrusive load monitoring
NN	Neural network
PCA	Principal component analysis
PDF	Probability density function
PReLU	Parametric rectified linear unit
PSD	Power spectral density
SCSAE	Stacked convolutional sparse auto-encoder
STFT	Short-time Fourier transform
SVD	Singular value decomposition
SVM	Support vector machine
T-SVD	Truncated singular value decomposition
